# Swine influenza virus infection in different age groups of pigs in farrow-to-finish farms in Thailand

**DOI:** 10.1186/1743-422X-8-537

**Published:** 2011-12-14

**Authors:** Nobuhiro Takemae, Sujira Parchariyanon, Ruttapong Ruttanapumma, Yasuaki Hiromoto, Tsuyoshi Hayashi, Yuko Uchida, Takehiko Saito

**Affiliations:** 1 Thailand-Japan Zoonotic Diseases Collaboration Center, Kasetklang, Chatuchak, Bangkok 10900, Thailand; 2 Research Team for Zoonotic Diseases, National Institute of Animal Health, National Agriculture and Food Research Organization (NARO), 3-1-5 Kannondai, Tsukuba, Ibaraki 305-0856, Japan; 3 National Institute of Animal Health, Kasetklang, Chatuchak, Bangkok, 10900, Thailand

**Keywords:** Influenza virus, Pig, Surveillance, Farrow-to-finish pig farm

## Abstract

**Background:**

Understanding swine influenza virus (SIV) ecology has become more and more important from both the pig industry and public health points of views. However, the mechanism whereby SIV occurs in pig farms is not well understood. The purpose of this study was to develop a proper strategy for SIV surveillance.

**Findings:**

We conducted longitudinal monitoring in 6 farrow-to-finish farms in the central region of Thailand from 2008 to 2009. Nasal swabs and serum samples were collected periodically from clinically healthy pigs consisting of sows, fattening pigs, weaned piglets and pigs transferred from other farms. A total of 731 nasal swabs were subjected to virus isolation and 641 serum samples were subjected to detection of SIV antibodies against H1 and H3 subtypes using the hemagglutination inhibition test and ELISA. Twelve SIVs were isolated in this study and eleven were from piglets aged 4 and 8 weeks. Phylogenetical analysis revealed that SIVs isolated from different farms shared a common ancestor. Antibodies against SIVs were detected in fattening pigs on farms with no SIV isolation in the respective periods studied. These observations suggested that piglets aged 8 weeks or younger could be a main target for SIV isolation. Farm-to-farm transmission was suggested for farms where pigs from other farms are introduced periodically. In addition, antibodies against SIVs detected in fattening pigs could be a marker for SIV infection in a farm.

**Conclusions:**

The present study provided important information on SIV surveillance that will enable better understanding of SIV ecology in farrow-to-finish farms.

## Background

Swine influenza virus (SIV) is one of the pathogens that cause respiratory diseases accompanied with coughing and sneezing in pigs [1]. This virus is considered an important pathogen not only from the viewpoint of animal health but also from that of public health [1-3]. Pigs can play the role of a 'mixing vessel' producing a novel influenza virus by genetic reassortment [4] as they have dual susceptibility to both human and avian influenza viruses [5]. Both receptors, namely, the sialic acid linked to galactose by an α2,6 linkage (SAα2,6Gal) for human viruses and an SAα2,3Gal for avian viruses, are expressed on epithelial cells of the tracheal and pulmonary structures of pigs [6,7]. The segmented nature of genomes of influenza A viruses allows the exchange of the gene segments when a pig is infected simultaneously with various viruses.

A novel H1N1 virus, later designated as a pandemic (H1N1) 2009 (H1N1pdm) virus, was first identified in April 2009 when it caused the first influenza pandemic in humans in the 21st century [8]. Origin of the NA and M gene segments of H1N1pdmv was found to be from an Eurasian avian-like H1N1 SIV while the remaining 6 segments were from a triple reassortant H1 SIV mainly circulating in North American swine [8]. Since it was discovered that H1N1pdmv is a reassortant between the two SIVs above, SIVs have attracted much attention from researchers worldwide.

Ecology of SIVs is highly complicated due to multiple genetic reassortments, although three subtypes H1N1, H1N2 and H3N2 are dominant in swine populations [1]. Avian-like H1N1 SIVs originally circulating among European pig populations have been found in China [9]. Triple reassortant H1N2 and H3N2 SIVs possessing genes from avian, human and swine viruses were found not only in North America [10,11] but also in South Korea [12] and Hong Kong [9]. World-wide dissemination of SIVs is considered to be linked with the transportation of breeding pigs. In addition, transmission of the H1N1pdmv from humans to domesticated animals, such as pigs in Argentina, South Korea and Canada [13-15], turkeys in Canada and Chile [16,17] and so on, has been demonstrated. Thus, viruses can generate novel genetic combinations that could arise anywhere in the world. A reassortant virus between H1N1pdmv and other SIVs has already been found in pig populations in Hong Kong at 9 months after the emergence of H1N1pdmv [9]. In such a situation, SIV control in a pig farm is crucial to prevent further genetic reassortment events in pigs that may trigger other pandemics in humans.

The pig industry in Thailand has been expanding rapidly as one of the major livestock industries since the 1970s [18]. Our previous study revealed that H1N1, H1N2 and H3N2 of SIVs circulated in Thailand from 2000 to 2005, and had acquired genetic diversity due to multiple introductions of classic swine, Eurasian avian-like swine and human viruses [19]. In addition, transmission of human viruses to pig [19] or vice versa [20] was also suggested. However, ecology and the prevalence of SIVs in the Thai pig population have not been well characterized. Here, we carried out longitudinal monitoring in farrow-to-finish farms in three provinces in the central region of Thailand from 2008 to 2009. Six farms consisting of two small family-operated farms, one middle sized farm and three large sized commercial farms were monitored. Both nasal swabs and serum samples were collected periodically from 4 different pig groups, namely, sow, fattening pigs, weaned piglets and pigs newly introduced into the farm. Virological and serological analyses in this study provided significant information needed to establish a strategy for SIV monitoring in farrow-to-finish farms.

## Materials and methods

### Collection of samples and epidemiological information

Forty nasal swabs were collected from 20 sows aged from 1 to 2 years, 10 fattening pigs aged 12 weeks and 10 weaned piglets aged 9 weeks in Farm A in January 2008. Five farms, B, C, D, E and F, were visited periodically to collect nasal swabs and blood samples three or more times from June 2008 to November 2009 (Table 1). Both samples were taken from 8 to 20 pigs in each of at least 3 different groups from farms B-F. Each group consisted of sows aged at least one year, fattening pigs aged from 3 to 4 months, and weaned piglets aged from 4 to 10 weeks. Specimens were also collected from pigs transferred from other farms to Farm B, C or E at the age of 8 weeks to 1 year since January 2009. Only nasal swabs were collected in Farm A in January 2008 and in Farm D in June 2008. Ten blood samples and twenty nasal swabs were collected from 20 pigs introduced in Farm B in September and November 2009. The sample size for each group allowed the detection of at least one positive pig at 95% confidence limits if the prevalence in each group exceeded 20-30% [21]. Epidemiological information of each farm was obtained by interviewing the farmers as listed in Table 1.

**Table 1:** lnformation on farrow-to-finish farms surveyed in this study

Farm	A	B	C	D	E	F
Sampling date	2008/1/29^d^	2008/6/9, 10/1, 2009/1/14, 7/1, 11/18	2008/6/23, 10/13, 2009/1/16, 7/2, 11/20	2008/6/16^d^, 10/6, 2009/1/15, 7/3, 11/19	2008/7/4, 11/15, 2009/1/8	2008/11/10, 2009/1/9, 7/10

Province	Ratchaburi	Saraburi	Saraburi	Saraburi	Singburi	Singburi

Number of pigs^a^: Sow	4000	2200	1100	120	20	20

Boar	100	12	30	30	1	1

Fattening	6000	12000	4900	160	60	60

Piglet^b^	6000	5700	2900	200	40	70

Purchase (Introduction of pigs from other farm or company)	A few pigs every few years	Piglet for breeding (8-wk-old): 20-25/week	Boar: 1-2/month Gilt(5,6-month-old): 50/week	20 boars in 2004	A few boars and sows in 2006, Three sows in 2009	A few sows in 2003, One boar in 2005

Purchased from:	Domestic farm	Domestic farm	Domestic farm	Denmark	Domestic farm	Domestic farm

Shower-in facilities for car/human	Yes/Yes	Yes/Yes	Yes/No	No/No	No/No	No/No

Presence of other domestic animals	Dog, cat	Dog	No	No	Cattle, dog, cat, crocodile	Chicken, dog

Period (age; week-old) for^c^:						

suckling stage	0-3	0-3	0-4	0-4	0-3	0-4(6)

weaning stage	3-9	3-8	4-11	4-8	3-6	4(6)-8

fattening stage	9-24	8-24	11-24	8-24	6-24	8-24

Vaccination for:	Aujeszky's disease (AD), Foot and Mouth disease (FMD), Parvo virus (PV), Swine fever (SF)	AD, Atrophic rhinitis (AR), FMD, Porcine reproductive and respiratory syndrome (PRRS), Porcine circovirus (PCV), Mycoplasma	AD, FMD, PRRS, PV, SF, Leptospirosis	FMD, PV, SF	AD, PRRS, SF, Mycoplasma	SF

^a^Averaged numbers throughout our surveillance

^b^'Piglet' was defined as a group of pigs in suckling and weaning stages

^c^Production system for rearing piglets

^d^Serum were not collected

### Virus isolation and phylogenetical analysis

All the nasal swabs were subjected to virus isolation at the National Institute of Animal Health (NIAH), Thailand as described previously [22]. Briefly, nasal swabs collected were immediately placed into a 15-ml tube containing 2 ml transport medium (MEM containing Penicillin (1000 unit/ml), Streptomycin (1000 μg/ml), Fungizone (25 μg/ml), 0.01 M HEPES and 0.5% bovine serum albumin). After centrifugation for 10 min at 2500 rpm, they were aliquoted. One portion was inoculated onto the monolayer of MDCK cells after filtering with a 0.45 μm pore size filter (Millipore, MA, USA). After viral adsorption to the cells, growth medium containing1 μg/ml of acetylated trypsin rather than fetal calf serum was added. If neither cytopathogenic effect nor HA activity with 1% guinea pig red blood cells was observed at 4 days after inoculation, the collected supernatant was inoculated in MDCK cells once more. Another portion including the nasal swab was subjected to viral RNA extraction using an RNeasy mini kit (Qiagen, Hilden, Germany), followed by RT-PCR using primers specific to either NP [23] or M [24]genes of the type A influenza virus. Subtypes were identified by the PCR method using specific primers designed in a previous study [19].

Direct sequencing of the PCR products and phylogenetic analysis of the viruses isolated in this study were carried out as described previously [19].

### Serological analysis

Serum was obtained from the collected blood samples by centrifugation for 10 min at 3500 rpm. All of the serum samples were subjected to the hemagglutination inhibition (HI) test and ELISA. Antigens used for the HI tests were A/swine/Ratchaburi/NIAH550/2003 (H1N1; Cla), A/swine/Ratchaburi/NIAH101942/2008 (H1N1; Clb), A/swine/Saraburi/NIAH107725-28/2008 (H3N2; Ha), and A/swine/Chachoengsao/2003 (H3N2; Hb) [19] and A/swine/Iowa/15/1930 (H1N1; Iowa), A/swine/Saraburi/NIAH116627-24/2009 (H1N1pdm) (Table 2). Serum samples for the HI test were treated overnight with receptor-destroying enzyme (RDE) from *Vibrio Cholerae *(Denka Seiken Co., Ltd., Tokyo, Japan) to remove any non-specific inhibitors of hemagglutination, and were then inactivated at 56°C for 30 min. Next, the serum samples were absorbed with packed chicken red blood cells for 60 min at room temperature. A cut off value of 1:40 was adopted to avoid false-positive cases due to non-specific reactions in the HI tests. Commercial ELISA kits (The HerdChek Swine Influenza H1N1 Antibody Test Kit and HerdChek Swine Influenza H3N2 Antibody Test Kit, IDEXX LABORATORIES, Inc., Maine, USA) were used to detect antibodies against 'classical' H1 and 'human-like' H3 SIV HAs according to the manufacturer's instructions.

**Table 2:** Swine influenza viruses isolated throughout the surveillance

Virrus	subtype	farm	isolated from (age):	Sampling date	Gene constellation^a^							
					
					HA	NA	PB2	PB1	PA	M	NP	NS
A/swine/Ratchaburi/NIAH101942/2008	H1N1	A	Fattening pig (12-wks)	2008/1/29	Clb	Ala	Ala	Ala	Alb	Alb	Ala	Ala

A/swine/Saraburi/NIAH100761-22/2009												
												
A/swine/Saraburi/NIAH100761-23/2009^b^	H1N1	B	Weaned piglet (4-wks)	2009/1/14	-	-	-	-	-	-	-	-
												
A/swine/Saraburi/NIAH100761-26/2009^b^												

A/swine/Saraburi/NIAH116625-11/2009												
					
A/swine/Saraburi/NIAH116625-12/2009^b^												
					
A/swine/Saraburi/NIAH116625-13/2009^b^	H1N1	B	Weaned piglet (8-wks)	2009/11/18	-	-	-	-	-	-	-	-
					
A/swine/Saraburi/NIAH116625-16/2009^b^												
					
A/swine/Saraburi/NIAH116625-17/2009^b^												

A/swine/Saraburi/NIAH107725-28/2008	H3N2	B	Weaned piglet (4-wks)	2008/6/9	Ha	Ha	-	-	Ala	Ala	Cla	Cla

A/swine/Saraburi/NIAH109713-36/2009	H3N2	B	Introduced pig (8-wks)	2009/7/1	Ha	Ha	-	-	Ala	Ala	Cla	Cla

A/swine/Saraburi/NIAH116627-24/2009	H1N1	D	Weaned piglet (8-wks)	2009/11/15	H1N1pdm origin^c^							

^a^Phylogenetic origins that are differrent from A/swine/Ratchaburi/NIAH101942/2008 are shown. Abbreviations are according to Takemae et al., (2008). Cl, AL and H stand for classical swine, avian-like swine and human origins, respectively. The small characters 'a' and 'b' after the origins show different clusters

^b^Phylogenetical origins of internal genes were determined by partial sequences

^c^All of genes of A/swine/Saraburi/NIAH116627-24/2009 were derived from pandemic (H1N1) 2009 (H1N1pdm) viruses

### Nucleotide sequence accession numbers

The sequences determined in this study are available from GenBank under accession numbers AB620160- AB620211.

## Results

### Epidemiological observations of the farms surveyed

**Figure 1:**
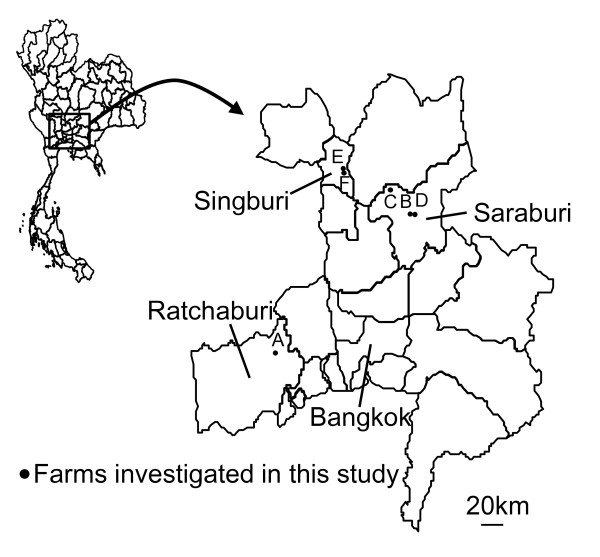
**Geographical location of the provinces where the surveillance was conducted in this study**.

A total of 731 nasal swabs and 641 serum samples were collected from six farms in the Ratchaburi, Saraburi and Singburi provinces in the central region of Thailand (Figure 1). All specimens were collected from clinically healthy pigs without influenza-like symptoms. There had been no movement of pigs between the farms investigated. Distance to the nearest pig farm from Farm A was 200 meters and that from Farm B was approximately 5 km. Owners of Farms C, D, E and F claimed that no pig farm existed in their vicinity. Total numbers of pigs in each farm on average ranged from 121 to 20,000. All of the farms visited were farrow-to-finish operated with pigs that are bred and fattened in each farm (Table 1). Pigs born in each farm are moved at least three times during their lifetime. Piglets were weaned from sows at 3 to 4 weeks old, and the weaning stage at the nursery house was until they were 6 to 11 weeks old (Table 1). After the fattening stage, they were sent to the slaughter houses at approximately 24 weeks old. Sows and newly introduced gilts were of Yorkshire-Landrace crosses, on the other hand, boars were Duroc in all of the farms. Farms A, B and C introduced pigs from domestic farms periodically, whereas D, E and F seldom did. Farm A introduces a few pigs as sows every few years while Farm B introduces 20-25 female breeding pigs at the age of 8 weeks every week, and Farm C about 50 pigs at the age of 5 to 6-months every week. The pigs are kept in quarantine piggeries for approximately 3 or 4 months in Farms B and C. After the absence of clinical signs is confirmed, they are moved to the breeding piggery for farrow. Farm D purchased 20 boars in 2004 from Denmark. Farms A and B had shower-in facilities for the entry of both cars and humans into the farms, and Farm C had such facilities for cars only. Domesticated animals other than pigs were kept on Farms A, B, E and F. Most of the piggeries in the farms were open or half-open providing easy access to wild birds and animals. In Farm B, porcine reproductive and respiratory syndrome (PRRS) occurred in piglets in the nursery house one week prior to the sampling on November 18, 2009. There was no report of respiratory diseases in pigs other than the incident in Farm B throughout the period of our monitoring. Vaccination was given as shown in Table 1 and that against swine influenza was not used in the farms investigated.

### Virus isolation

**Figure 2:**
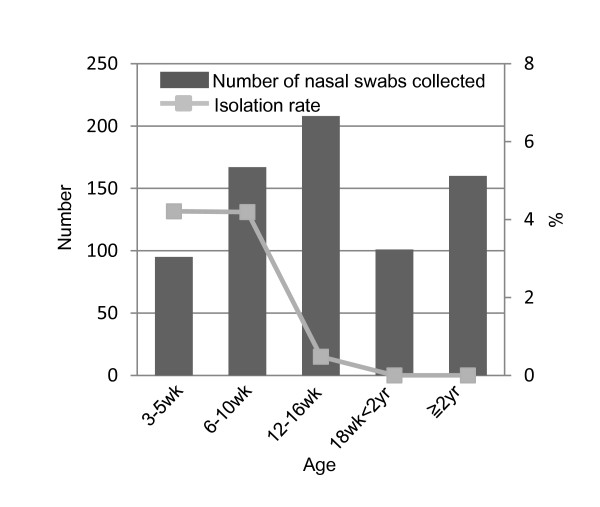
**Age distribution of the numbers of nasal swabs collected and isolation rate of swine influenza viruses**. Bar graph shows the numbers of nasal swabs collected in this study. Solid line shows the isolation rate of swine influenza viruses.

Twelve viruses consisting of 10 H1N1 SIVs and 2 H3N2 SIVs were isolated from 731 swabs collected (Table 2). Total virus isolation rate was 1.6% (4.2% in piglets aged from 3 to 5 weeks, 4.2% in piglets aged from 6 to 10 weeks, 0.5% in fattening pigs aged from 12 to 16 weeks, 0% in pigs aged more than 18 weeks) (Figure 2). Pigs proven to be infected with SIVs by virus isolation were 4 to 12 weeks old. All of the nasal swabs yielding viruses in MDCK cells were positive by conventional PCR with influenza specific primers (NP or M). Viruses were isolated after 3-4 days incubation following the inoculation of each swab except one. The excluded swab required a second passage in MDCK and the virus titer in the original swab was 10^0.8 ^TCID_50_/ml, which was the lowest TCID_50_/ml of the swabs that yielded viruses in this study.

Nine out of 10 H1N1 viruses isolated throughout the study appeared to share a common ancestor with the Thai SIVs identified in our previous study [19]. A/swine/Ratchaburi/NIAH101942/2008 (H1N1) (Rat101942) was isolated from a 12-week old fattening pig in Farm A [22]. Three H1N1 SIVs, designated as A/swine Saraburi/NIAH100761-22/2009 (H1N1) (Sara100761-22), A/swine/Saraburi/NIAH100761-23/2009 (H1N1), and A/swine/Saraburi/NIAH100761-26/2009 (H1N1), were isolated from 4-week-old weaned piglets kept in the same compartment on January 14,2009 in Farm B (Table 2). Five H1N1 SIVs were also isolated in Farm B from 8-week-old weaned piglets on November 18, 2009. They were designated as A/swine/Saraburi/NIAH116625-11/2009 (H1N1) (Sara116625-11), A/swine/Saraburi/NIAH116625-12/2009 (H1N1), A/swine/Saraburi/NIAH116625-13/2009 (H1N1), A/swine/Saraburi/NIAH116625-16/2009 (H1N1), and A/swine/Saraburi/NIAH116625-17/2009 (H1N1). Rat101942 shared more than 98.2% and 97.6% nucleotide identities with Sara100761-22 and Sara116625-11 in each segment, respectively. Sara100761-22 and Sara11625-11 shared more than 99.4% nucleotide identities in all of the eight gene segments. Based on the phylogenetic analyses of all gene segments, the gene constellation was similar to those of Thai SIVs isolated from 2004 to 2005 and represented by A/swine/Chonburi/NIAH589/2005 (H1N1) and A/swine/Chachoengsao/NIAH587/2005 (H1N1) in our previous study [19] (Additional file 1: Supplementary Figures S1-S3). HA genes of the current isolates belonged to the Clb cluster of the classical swine lineage (Additional file 1: Supplementary Figure S1), while PA (Additional file 1: Supplementary Figure S2) and M genes to the ALb cluster and NA, PB2, PB1, NP and NS (Additional file 1: Supplementary Figure S3) genes belonged to ALa within the Eurasian avian-like swine lineage (Table 2). A/swine/Saraburi/NIAH116627-24/2009 (H1N1) (Sara116627-24) was isolated in Farm D from a weaned piglet at the age of 8 weeks (Table 2). Sequencing analysis confirmed that all of the eight gene segments of Sara116627-24 originated from H1N1pdmv (Table 2).

Two H3N2 SIVs, A/swine/Saraburi/NIAH107725-28/2008 (H3N2) (Sara107725-28) and A/swine/Saraburi/NIAH109713-36/2009 (H3N2) (Sara109713-36), were isolated from Farm B. Sara107725-28 was isolated from a weaned piglet at the age of 4 weeks on June 9, 2008. Sara109713-36 was isolated from an 8-week-old introduced piglet on July 1, 2009. They shared high nucleotide homologies of more than 96.7% in each segment and their gene constellations were similar and identical with that of A/swine/Ratchaburi/NIAH874/05 (H3N2), reported elsewhere [19] (Additional file 1: Supplementary Figures S2-S4). The HA (Additional file 1: Supplementary Figure S4) and NA genes belonged to the Ha cluster, which is one of the two distinct human-like Thai SIV clusters existing within the human H3N2 lineages [19]. NP and NS (Additional file 1: Supplementary Figure S3) genes belonged to Cla which is a different cluster from Clb formed by Thai isolates within a classical swine lineage [19]. The remaining genes were clustered in an ALa sub-cluster of Eurasian avian-like SIVs (Table 2).

### Serologic results of farms surveyed in this study

In the analysis of sero-reactivities of the collected serum against the H1 subtype, different reactivities were observed between the ELISA and HI tests (Table 3). Positive rate in the ELISA was equal or higher than that obtained for the HI tests with 4 different antigens in most of the cases. For the serum collected from sows in Farm B in June 2008, however, positive rates against Cla, Clb and Iowa were higher than those with the ELISA. Also, for the serum collected in Farm E in January 2009, higher rates were observed with Cla, Clb and Iowa as the antigens. Positive reactions against the H1N1pdm antigen were observed in Farms C and E even before the virus appeared in the human population. However, it seemed that those reactions were most likely due to cross-reactions with the antigen, since the positive rates were always less than those against other antigens except in Farm D where H1N1pdmv was indeed isolated (Table 3).

**Table 3:** Number of seropositive pigs H1 swine influenza viruses by commercial H1N1 Swine influenza virus ELISA test and HI test in different farms and different age group

Farm	Group	Jun.-Jul. 2008						Oct.-Nov. 2008						Jan. 2009						Jul. 2009						Nov. 2009					
		
		ELISA^a^	HI^b^				*n^c^*	ELISA	HI				*n*	ELISA	HI				*n*	ELISA	HI				*n*	ELISA	HI				*n*
																
			Cla	Clb	lowa	Pdm			Cla	Clb	lowa	Pdm			Cla	Clb	lowa	Pdm			Cla	Clb	lowa	Pdm			Cla	Clb	lowa	Pdm	
B	Sow	6	9	10	7	0	*10*	3	2	1	1	0	*10*	3	2	3	1	0	*10*	7	1	3	3	1	*10*	6	3	6	2	3	*10*
	
	Fattening pig	*-^d^*						3	2	4	0	0	*10*	1	0	0	0	0	*10*	6	2	4	0	0	*10*	0	0	0	0	0	*10*
	
	Weaned pig	4	1	2	1	0	*20*	4	3	1	0	0	*10*	2	3	0	0	0	*10^e^*	2	0	0	0	0	*10*	0	0	0	0	0	*10^e^*
	
	Introduced pig	-						-						-						0	0	0	0	0	*10*	0	0	0	0	0	*10*

C	Sow	8	6	6	4	3	*10*	9	8	6	6	5	*10*	9	7	7	5	1	*10*	7	4	3	4	1	*10*	10	9	9	9	6	*10*
	
	Fattening pig	1	1	0	0	0	*10*	2	1	0	0	0	*10*	5	2	0	0	0	*10*	7	0	2	0	0	*10*	1	0	0	0	0	*10*
	
	Weaned pig	1	0	0	0	0	*10*	0	0	0	0	0	*10*	0	0	0	0	0	*10*	0	0	0	0	0	*10*	0	0	0	0	0	*10*
	
	Introduced pig	-						-						-						5	2	0	1	0	*10*	8	4	6	6	2	*11*

D	Sow	-						0	0	0	0	0	*10*	0	0	0	0	0	*10*	0	0	0	0	0	*10*	10	10	9	6	10	*10*
								
	Fattening pig							0	0	0	0	0	*10*	0	0	0	0	0	*10*	0	0	0	0	0	*10*	2	2	1	2	8	*10*
								
	Weaned pig							0	0	0	0	0	*10*	0	0	0	0	0	*10*	0	0	0	0	0	*10*	0	0	0	0	1	*10^e^*

E	Sow	2	1	1	1	1	*10*	1	1	0	0	1	*10*	4	4	5	5	3	*8*	-						-					
													
	Fattening pig	0	0	0	0	0	*10*	0	0	0	0	0	*10*	3	9	9	3	0	*10*												
													
	Weaned pig	0	0	0	0	0	*10*	0	0	0	0	0	*10*	0	4	8	4	0	*10*												
													
	Introduced pig	-						-						0	0	0	0	0	*3*												

F	Sow	-						0	0	0	0	0	*10*	0	0	0	0	0	*10*	0	0	0	0	0	*9*	-					
														
	Fattening pig							0	0	0	0	0	*10*	0	0	0	0	0	*10*	0	0	0	0	0	*10*						
														
	Weaned pig							0	0	0	0	0	*10*	0	0	0	0	0	*10*	0	0	0	0	0	*10*						

^a^Sample to positive (S/P) ratios were calculated using the optical density (OD) of each sample, positive control and negative control. The S/P ratios greater than 0.40 were considered as positive for antibodies againts H1N1 SlVs

^b^Samples showing HI activity at 1:40 or higher were considered as positve againts antigens A/swine/Ratchaburi/NIAH550/2003(H1N1)(Cla), A/swine/Ratchaburi/NIAH101942/2008(H1N1)(Clb), A/swine/lowal/15/1930 (H1N1)(lowa) and A/swine/Saraburi/NIAH112226-24/2009 (H1N1pdm)(Pdm)

^c^Number of serums collected

^d^Samples were not collected

^e^The groups from which H1N1 SIVs were isolated

In Farms B and C, some sows were always seropositive against H1 antigens whereas fattening and weaned pigs in Farm B did not show any positivity towards the H1 antigens in November 2009 (Table 3). Pigs introduced at the age of 8 weeks in Farm B did not show positivity towards any of the H1 antigens in July 2009 and November 2009. In Farm C, weaned pigs did not show any positivity towards the H1 antigens from October 2008 to November 2009. In contrast, pigs introduced in Farm C from 5 to 6 months of age in July 2009 and November 2009 showed positivity towards the H1 antigens.

In Farms D and F, no pig positive for the H1 antigens used was found until July 2009 (Table 3). All sera from sows collected in Farm D in November 2009 were positive in the HI test with Sara116627-24 (H1N1pdmv). At that time, 8 out of 10 fattening pigs and 1 out of 10 weaned piglets were also positive towards H1N1pdmv in Farm D.

In Farm E, the number of seropositive pigs apparently increased in January 2009 in all the groups (Table 3). Although only one or two sows were positive towards classical H1 before 2009, 3 to 5 out of 8 sows showed positivity in the ELISA and HI tests in January 2009. At that time, 9 out of 10 fattening pigs were positive towards Cla and Clb, and 3 of them were positive in the ELISA and HI test using Iowa. In addition, more than 4 weaned piglets were positive towards Cla, Clb and Iowa at the same time. Three pigs introduced at the age of 1 year at 1 day prior to the sampling in January 2009 were serologically negative towards classical H1 SIVs.

Clear contrast was seen in the reactivity of the serum against Ha and Hb viruses in the HI tests of the H3 subtype (Table 4). Seropositive pigs against the Hb virus were found in Farm C only, whereas pigs positive towards the Ha virus were found in Farms B, D, E and F and they were negative towards the Hb virus. No significant correlation between the reactivity with the ELISA and Ha or Hb viruses was seen, since in several occasions only positives towards the ELISA were observed.

**Table 4:** Number of seropositive pigs against H3 swine influenza viruses by commercial H3N2 Swine influenza virus ELISA test and HI test in different farms and different age groups

Farm	Groups	Jun.-Jul.2008				Oct.-Nov.2008				Jan.2009				Jul. 2009				Nov. 2009			
		
		ELISA^a^	HI^b^		*n^c^*	ELISA	HI		*n*	ELISA	HI		*n*	ELISA	HI		*n*	ELISA	HI		*n*
																
			Ha	Hb			Ha	Hb			Ha	Hb			Ha	Hb			Ha	Hb	
B	Sow	8	5	0	*10*	6	4	0	*10*	8	6	0	*10*	5	4	0	*10*	5	3	0	*10*
	
	Fattening pig	*- ^d^*				5	6	0	*10*	6	3	0	*10*	10	7	0	*10*	0	0	0	*10*
	
	Weaned pig	0	5	0	*20^e^*	2	1	0	*10*	0	1	0	*10*	2	0	0	*10*	0	0	0	*10*
	
	Introduced pig	*-*				*-*				*-*				1	3	0	*10^e^*	0	0	0	*10*

C	Sow	5	0	4	*10*	1	0	4	*10*	2	0	3	*10*	1	0	1	*10*	3	0	0	*10*
	
	Fattening pig	0	0	0	*10*	0	0	0	*10*	0	0	0	*10*	0	0	0	*10*	0	0	0	*10*
	
	Weaned pig	0	0	0	*10*	0	0	0	*10*	0	0	0	*10*	0	0	0	*10*	0	0	0	*10*
	
	Introduced pig	*-*				*-*				*-*				0	0	0	*10*	0	0	0	*11*

D	Sow	*-*				0	0	0	*10*	2	0	0	*10*	0	0	0	*10*	1	0	0	*10*
						
	Fattening pig					0	0	0	*10*	0	0	0	*10*	0	0	0	*10*	0	0	0	*10*
						
	Weaned pig					0	0	0	*10*	0	0	0	*10*	0	0	0	*10*	0	0	0	*10*

E	Sow	10	10	0	*10*	10	9	0	*10*	8	8	0	*8*	*-*				*-*			
									
	Fattening pig	0	0	0	*10*	2	0	0	*10*	0	0	0	*10*								
									
	Weaned pig	0	0	0	*10*	3	0	0	*10*	2	0	0	*10*								
									
	Introduced pig	*-*				*-*				0	0	0	*3*								

F	Sow	*-*				1	0	0	*10*	1	1	0	*10*	1	1	0	*9*	*-*			
										
	Fattening pig					0	0	0	*10*	0	0	0	*10*	0	0	0	*10*				
										
	Weaned pig					1	0	0	*10*	0	0	0	*10*	0	0	0	*10*				

^a^Sample to positve (S/P) ratios were calculated using the optical density (OD) of each sample, positive control and negative control. The S/P rations greater than 0.40 were considered as positive for antibodies against H3N2 SIVs

^b^Samples showing HI activity at 1:40 or higher were considered as positive against antigens A/swine/Saraburi107725-28/2008 (H3N2) (Ha) and A/swine/Chachoengsao/2003 (H3N2) (Hb)

^c^Number of serums collected

^d^Samples were not collected

^e^The groups from which H3N2 SIVs were isolated

In Farms B, C and E, as seen for the H1 serology, sows were positive towards the H3 antigens in all occasions, whereas fattening and weaned pigs in Farm B were negative in November 2009 (Table 4). Fattening and weaned pigs were negative at all occasions in Farm C. In Farm E, the tests were negative in June 2008. A few sows were found positive by ELISA twice during the surveillance in Farm D. In Farm F, sows were positive on three occasions by ELISA and/or the HI test with the Ha antigen and one weaned pig was positive by ELISA in October 2008.

## Discussion

The mechanisms of SIV introduction in farrow-to-finish pig farms in Thailand have not been well studied. In this study, we conducted a longitudinal surveillance in farrow-to-finish pig farms located in the central part of Thailand to develop a proper strategy for SIV surveillance. We found that young pigs, in particular, piglets at the age of 8 weeks or younger could be the target animals to isolate SIVs circulating in farms. Seroprevalence against SIVs in fattening pigs was evidence that SIV infection did occur within farms, while results of a phylogenetical analysis suggested that farm-to-farm transmission had occurred. In addition, a discrepancy between the HI test and ELISA suggested the possibility that the sub-lineages of H1 and H3 SIVs that have not yet been isolated may be circulating in the Thai pig population. Thus, information obtained in this study would be useful for conducting SIV surveillances in farrow-to-finish farms.

In this study, SIVs were most frequently isolated from weaned piglets aged 4 and 8 weeks. Previous findings also pointed out that the majority of SIV infections take place in piglets aged under 10 weeks [25]. Weaned piglets are considered to be susceptible to SIVs because the concentration of the maternal antibodies against SIVs in the serum declines with age in piglets [26], and the half life of antibodies against H1 and H3 SIVs was estimated to be 12 days [25]. The high density of pigs in piggeries and large size herds is a contributing factor to the high SIV prevalence rates in fattening pigs and sows [27,28]. Thus, gathering of weaned piglets in one nursery together with other piglets farrowed from different sows is another likely factor contributing to the high isolation rate in weaned piglets in farrow-to-finish farms.

Dissemination of the SIV of a particular genotype was suggested based on the fact that the H1N1 SIVs isolated from Farms A and B shared a common ancestor. Pigs and other materials were not transferred between farms and moreover, the farms were separated geographically by more than 100 km, suggesting that SIVs have spread extensively in Thailand. The introduction of pigs carrying SIVs is one of the most likely factors for viral dissemination among farms [1]. The isolation of Sara109713-36 from a pig introduced to Farm B may have originated from the farm from which this pig was introduced. At the same time, there remains a possibility that the pig was infected after being introduced to the farm, because the affected pig was introduced into Farm B 4 days prior to the sampling date. A period of 4 days is known to be enough for pigs to start virus shedding after experimental infection [29]. The other pigs introduced earlier than the affected pig were also in the same quarantined piggery, although they were separated into different compartments. In addition, there were no regulations for the movement of humans between piggeries (quarantine piggery, breeding/farrowing sites, weaned sites and fattening sites) in that farm.

Serological analysis revealed that the detection of antibodies against SIVs in fattening pigs could be an indicator of SIV infection in a farrow-to-finish farm. Maternal antibodies declined in fattening pigs aged 3 to 4 months [26,30]. In addition, fattening pigs were replaced with neonatal pigs at each sampling in this study. Thus, fluctuations in the seropositive rate observed in fattening pigs indicated that SIV infection occurred prior to each sampling. On the other hand, neither the antibodies found in serum of weaned piglets nor those in sows could be used as an indicator of the recent SIV occurrence in a farm. Serological tests cannot distinguish maternal antibodies from those due to SIV infection. Sows are kept in a farm for more than a few years and antibodies against classical H1 SIVs in a pig are known to last up to more than 1 year after the primary infection [29]. Thus, detection of the antibodies cannot indicate a recent infection of the sows. In farms such as Farms B and C where gilts are frequently introduced, it is not clear whether the seropositive sow was infected with SIVs before or after it was introduced into the farm.

The presence of seropositive fattening pigs in Farms C and E suggested SIV infection, however, no SIV was isolated. Sero-positive reaction was always observed against H1 SIVs in fattening pigs in Farm C, and an apparent increase in the number of fattening pigs seropositive against H1 SIVs was observed in January 2009 in Farm E. The reason why SIVs were not isolated in these farms may be explained by the fact that SIVs could circulate continuously in those farms with a prevalence rate lower than the detection limit rate in our sampling numbers. Sows were suggested to be a reservoir for continuous circulation of some respiratory pathogens (eg. *Actinobacillus pleuropneumoniae*, Porcine Circovirus type-2, SIVs) in a farm. Antibodies against those pathogens were detected at high rates in the sow population in a farm [31]. Indeed, sows showed the highest seropositive rate in most samplings among the 4 groups examined in this study, suggesting the possibility that sows are repeatedly infected with SIVs during their 4 to 5 years of stay on a farm. Frequent introduction of pigs into a farm, such as Farm C, could also allow viral entry into the fattening pig population. In addition, movement of people/materials between farms could also be a possible route of entry of SIVs as was the case in Farm E where pigs were seldom introduced. Thus, further investigation is necessary to elucidate the mechanism of SIV persistence in farms.

Serological analysis suggested that SIVs belonging to unidentified sub-lineages within classical H1 and human-like H3 viruses likely exist in the Thai pig population. HI tests using various antigens revealed that antigenicity of the antigens within the subtypes in both H1 and H3 can vary. IDEXX ELISA often detected antibodies in serum samples that showed up negative in the HI tests. The antigens selected for use in the HI tests of this study represented SIVs circulating in Thailand according to our previous study [19]. The ELISA test appeared to detect antibodies that could not be detected by the HI tests with the antigens used. This suggests that viruses possessing antigenicities different from those of the SIVs used in this study may be circulating in the Thai pig population.

H1N1pdmv infection in pigs in Thailand was detected in Farm D during our longitudinal monitoring. Direct human to pig transmission was suspected as was the case in pig farms in other countries [13,32], because the affected farm had not introduced pigs since 2004. Based on the serological results, there is the possibility that H1N1pdmv was first introduced into sows before November 2009, and it then spread to fattening pigs and piglets within the farm. In Thailand, the number of confirmed human cases of H1N1pdmv infection increased from 8,800 to 28,300 from the end of June to October 2009 [33]. Until July 3, 2009, the affected farm was shown to be free of H1N1pdmv by retrospective serological analysis. Remarkably, no significant clinical symptom was observed in the piglets carrying the virus at the time of swab collection, which is unlike other H1N1pdmv infections in pigs that have been reported along with respiratory symptoms [13,32]. Therefore, the actual number of H1N1pdmv cases in the pig population may be much higher than that reported worldwide. According to the OIE weekly diseases information, up to March 2011, H1N1pdmv infection in pigs had been reported in 21 countries/districts [34]. Emergence of reassortants with H1N1pdmv and other SIVs in pigs could be a threat to public health [35,36]. In addition, transmission of H1N1pdmv from humans to pigs could have caused the amino acid changes in the HA, NA, M and NP genes, suggesting the possibility of a significant impact on viral evolution [37]. Thus, to minimize the risk of H1N1pdmv infection in pigs, extensive bio-security protocols for farms need to be considered.

Many researchers have pointed out the importance of monitoring SIVs because pigs have the potential role as a mixing vessel for influenza viruses [1,4]. To date, highly pathogenic H5N1 avian influenza viruses have been isolated sporadically in China [38] and Indonesia [39]. H9N2 viruses that infect not only poultry but also humans [40,41] were isolated from pigs from 1998 to 2007 in China. Under such circumstances, it is important that knowledge on the occurrence of SIVs in farms be deepened. The information obtained in this study could be useful to develop a strategy for SIV surveillance not only in Thailand but also in other countries, since the farrow-to-finish production system is commonly conducted worldwide. Crucial factors that determine the persistence and infection of SIVs in farms remain unclear. Further studies on SIVs in farms are needed in order to prevent economical losses caused by these viruses, and to prevent the emergence of novel viruses with the potential to cause pandemics in humans.

## Conclusions

In the present study, we conducted SIV surveillance in 6 farrow-to-finish farms in the central part of Thailand from 2008 to 2009. Twelve SIVs including 10 H1N1 and 2 H3N2 subtypes were isolated from 731 nasal swabs. All of the SIVs were isolated solely from young pigs aged from 4 to 12 weeks in the farrow-to-finish farms surveyed. Meanwhile, from the serological analyses, the seroprevalence against SIVs observed in fattening pigs showed evidence of recent SIV occurrence even in the farms where no SIVs had been isolated. Thus, these two findings could be potent tools for conducting SIV surveillances in farrow-to-finish farms.

## List of Abbreviations

SIV: Swine influenza virus; SAα2,6Gal: Sialic acid linked to galactose by α2,6 linkage; SAα2,3Gal: Sialic acid linked to galactose by α2,3 linkage; H1N1pdm: pandemic (H1N1) 2009; HI test: hemagglutination inhibition test; TCID_50_: 50% tissue culture infective dose; Rat101942: A/swine/Ratchaburi/NIAH101942/2008 (H1N1); Sara100761-22: A/swine Saraburi/NIAH100761-22/2009 (H1N1); Sara116625-11: A/swine/Saraburi/NIAH116625-11/2009 (H1N1); Sara116627-24: A/swine/Saraburi/NIAH116627-24/2009 (H1N1); Sara107725-28: A/swine/Saraburi/NIAH107725-28/2008 (H3N2); Sara109713-36: A/swine/Saraburi/NIAH109713-36/2009 (H3N2)

## Competing interests

The authors declare that they have no competing interests.

## Authors' contributions

NT, YH, TH and YU carried out virus isolation from the specimens. NT carried out genetical and serological analyses. SP and RR coordinated the pig farm visits. All authors participated in the sampling specimens at the farms. NT and TS designed the study and draft the manuscript. All authors read and approved the final manuscript.

## Supplementary Material

Additional file 1 Supplementary figure S1; Supplementary figure S2; Supplementary figure S3; Supplementary figure S4 Click here for file
