# Supplementary material for: Swine influenza virus infection in different age groups of pigs in farrow-to-finish farms in Thailand
Source: Virol J. 2011 Dec 14;8:537. doi: 10.1186/1743-422X-8-537 (PMC3308982; doi:10.1186/1743-422X-8-537)
Supplement: Additional file 1 — Supplementary figure S1; Supplementary figure S2; Supplementary figure S3; Supplementary figure S4 [file 1743-422X-8-537-S1.PPT]

## Slide 1
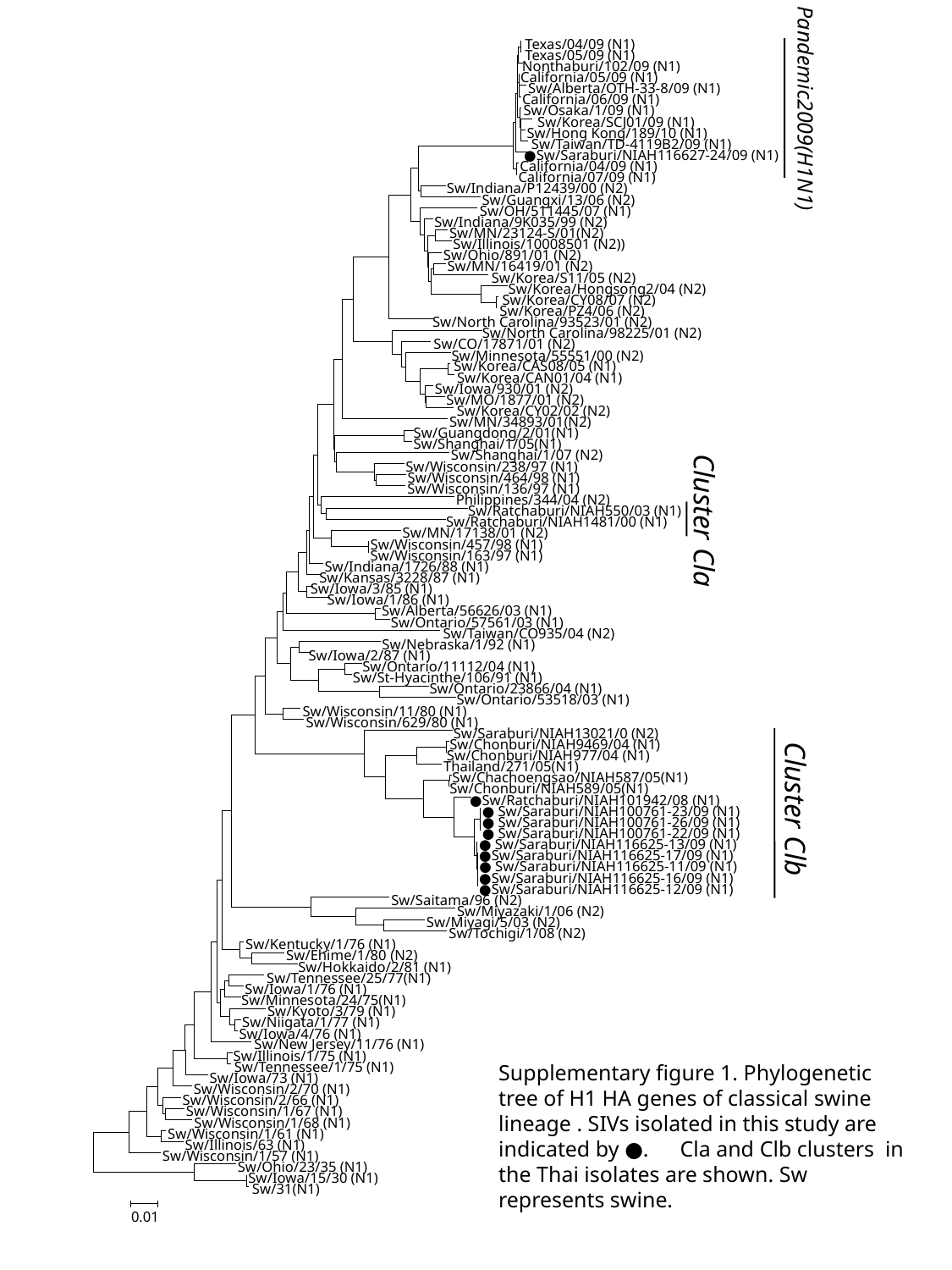

Texas/04/09 (N1)
 Texas/05/09 (N1)
 Nonthaburi/102/09 (N1)
 California/05/09 (N1)
 Sw/Alberta/OTH-33-8/09 (N1)
 California/06/09 (N1)
 Sw/Osaka/1/09 (N1)
 Sw/Korea/SCJ01/09 (N1)
 Sw/Hong Kong/189/10 (N1)
 Sw/Taiwan/TD-4119B2/09 (N1)
●Sw/Saraburi/NIAH116627-24/09 (N1)
 California/04/09 (N1)
 California/07/09 (N1)
 Sw/Indiana/P12439/00 (N2)
 Sw/Guangxi/13/06 (N2)
 Sw/OH/511445/07 (N1)
 Sw/Indiana/9K035/99 (N2)
 Sw/MN/23124-S/01(N2)
 Sw/Illinois/10008501 (N2))
 Sw/Ohio/891/01 (N2)
 Sw/MN/16419/01 (N2)
 Sw/Korea/S11/05 (N2)
 Sw/Korea/Hongsong2/04 (N2)
 Sw/Korea/CY08/07 (N2)
 Sw/Korea/PZ4/06 (N2)
 Sw/North Carolina/93523/01 (N2)
 Sw/North Carolina/98225/01 (N2)
 Sw/CO/17871/01 (N2)
 Sw/Minnesota/55551/00 (N2)
 Sw/Korea/CAS08/05 (N1)
 Sw/Korea/CAN01/04 (N1)
 Sw/Iowa/930/01 (N2)
 Sw/MO/1877/01 (N2)
 Sw/Korea/CY02/02 (N2)
 Sw/MN/34893/01(N2)
 Sw/Guangdong/2/01(N1)
 Sw/Shanghai/1/05(N1)
 Sw/Shanghai/1/07 (N2)
 Sw/Wisconsin/238/97 (N1)
 Sw/Wisconsin/464/98 (N1)
 Sw/Wisconsin/136/97 (N1)
 Philippines/344/04 (N2)
 Sw/Ratchaburi/NIAH550/03 (N1)
 Sw/Ratchaburi/NIAH1481/00 (N1)
 Sw/MN/17138/01 (N2)
 Sw/Wisconsin/457/98 (N1)
 Sw/Wisconsin/163/97 (N1)
 Sw/Indiana/1726/88 (N1)
Sw/Kansas/3228/87 (N1)
Sw/Iowa/3/85 (N1)
Sw/Iowa/1/86 (N1)
 Sw/Alberta/56626/03 (N1)
 Sw/Ontario/57561/03 (N1)
 Sw/Taiwan/CO935/04 (N2)
 Sw/Nebraska/1/92 (N1)
Sw/Iowa/2/87 (N1)
 Sw/Ontario/11112/04 (N1)
 Sw/St-Hyacinthe/106/91 (N1)
 Sw/Ontario/23866/04 (N1)
 Sw/Ontario/53518/03 (N1)
 Sw/Wisconsin/11/80 (N1)
 Sw/Wisconsin/629/80 (N1)
 Sw/Saraburi/NIAH13021/0 (N2)
 Sw/Chonburi/NIAH9469/04 (N1)
 Sw/Chonburi/NIAH977/04 (N1)
 Thailand/271/05(N1)
 Sw/Chachoengsao/NIAH587/05(N1)
 Sw/Chonburi/NIAH589/05(N1)
 ●Sw/Ratchaburi/NIAH101942/08 (N1)
 ● Sw/Saraburi/NIAH100761-23/09 (N1)
 ● Sw/Saraburi/NIAH100761-26/09 (N1)
 ● Sw/Saraburi/NIAH100761-22/09 (N1)
 ● Sw/Saraburi/NIAH116625-13/09 (N1)
 ●Sw/Saraburi/NIAH116625-17/09 (N1)
 ● Sw/Saraburi/NIAH116625-11/09 (N1)
 ●Sw/Saraburi/NIAH116625-16/09 (N1)
 ●Sw/Saraburi/NIAH116625-12/09 (N1)
 Sw/Saitama/96 (N2)
 Sw/Miyazaki/1/06 (N2)
 Sw/Miyagi/5/03 (N2)
 Sw/Tochigi/1/08 (N2)
 Sw/Kentucky/1/76 (N1)
 Sw/Ehime/1/80 (N2)
 Sw/Hokkaido/2/81 (N1)
 Sw/Tennessee/25/77(N1)
 Sw/Iowa/1/76 (N1)
 Sw/Minnesota/24/75(N1)
 Sw/Kyoto/3/79 (N1)
 Sw/Niigata/1/77 (N1)
 Sw/Iowa/4/76 (N1)
 Sw/New Jersey/11/76 (N1)
 Sw/Illinois/1/75 (N1)
 Sw/Tennessee/1/75 (N1)
 Sw/Iowa/73 (N1)
 Sw/Wisconsin/2/70 (N1)
 Sw/Wisconsin/2/66 (N1)
 Sw/Wisconsin/1/67 (N1)
 Sw/Wisconsin/1/68 (N1)
 Sw/Wisconsin/1/61 (N1)
 Sw/Illinois/63 (N1)
 Sw/Wisconsin/1/57 (N1)
 Sw/Ohio/23/35 (N1)
 Sw/Iowa/15/30 (N1)
 Sw/31(N1)
0.01
Pandemic2009(H1N1)
Cluster Cla
Cluster Clb
Supplementary figure 1. Phylogenetic tree of H1 HA genes of classical swine lineage . SIVs isolated in this study are indicated by ●.　Cla and Clb clusters in the Thai isolates are shown. Sw represents swine.

## Slide 2
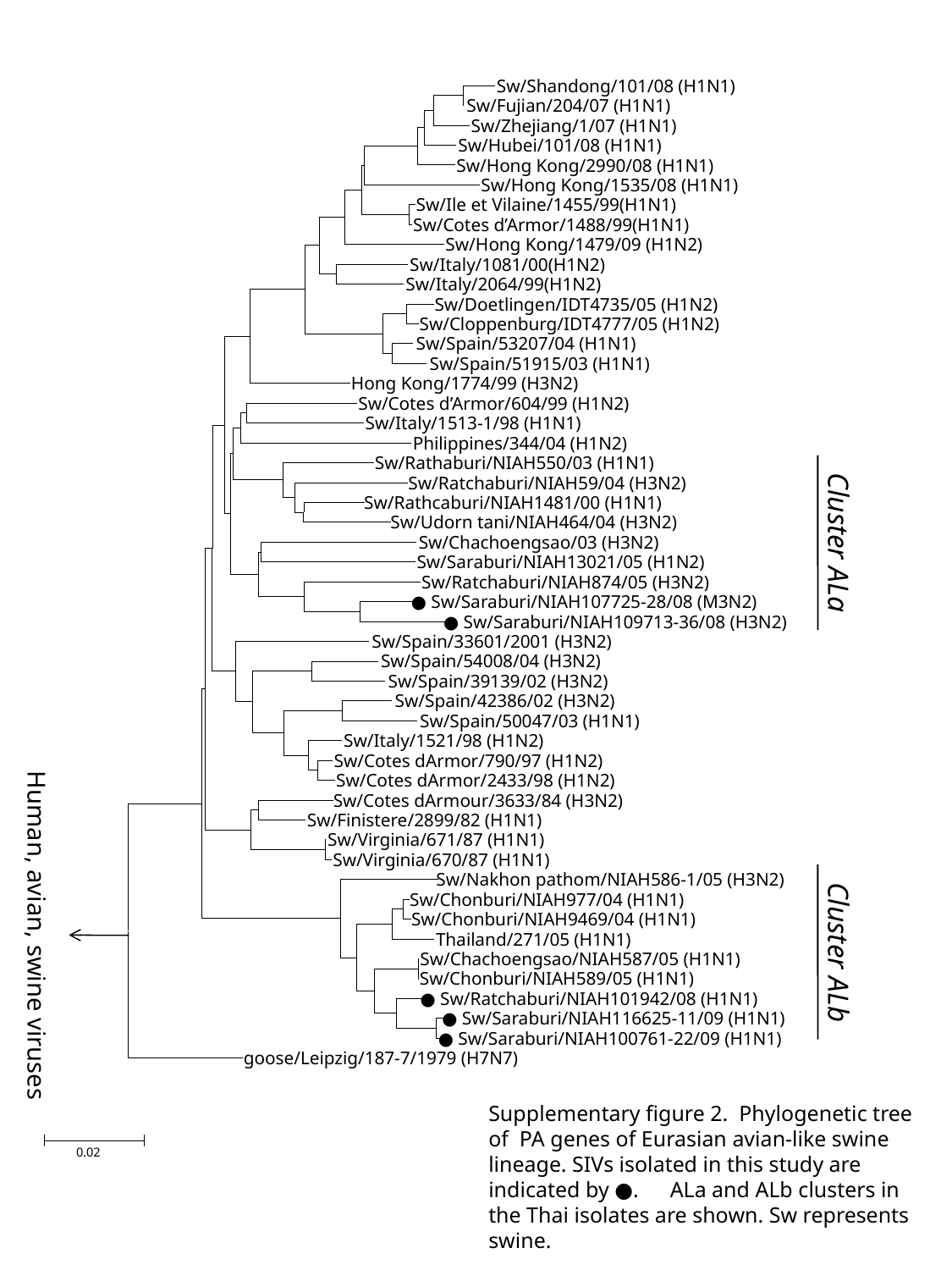

Sw/Shandong/101/08 (H1N1)
 Sw/Fujian/204/07 (H1N1)
 Sw/Zhejiang/1/07 (H1N1)
 Sw/Hubei/101/08 (H1N1)
 Sw/Hong Kong/2990/08 (H1N1)
 Sw/Hong Kong/1535/08 (H1N1)
 Sw/Ile et Vilaine/1455/99(H1N1)
 Sw/Cotes d’Armor/1488/99(H1N1)
 Sw/Hong Kong/1479/09 (H1N2)
 Sw/Italy/1081/00(H1N2)
 Sw/Italy/2064/99(H1N2)
 Sw/Doetlingen/IDT4735/05 (H1N2)
 Sw/Cloppenburg/IDT4777/05 (H1N2)
 Sw/Spain/53207/04 (H1N1)
 Sw/Spain/51915/03 (H1N1)
 Hong Kong/1774/99 (H3N2)
 Sw/Cotes d’Armor/604/99 (H1N2)
 Sw/Italy/1513-1/98 (H1N1)
 Philippines/344/04 (H1N2)
 Sw/Rathaburi/NIAH550/03 (H1N1)
 Sw/Ratchaburi/NIAH59/04 (H3N2)
 Sw/Rathcaburi/NIAH1481/00 (H1N1)
 Sw/Udorn tani/NIAH464/04 (H3N2)
 Sw/Chachoengsao/03 (H3N2)
 Sw/Saraburi/NIAH13021/05 (H1N2)
 Sw/Ratchaburi/NIAH874/05 (H3N2)
 ● Sw/Saraburi/NIAH107725-28/08 (M3N2)
 ● Sw/Saraburi/NIAH109713-36/08 (H3N2)
 Sw/Spain/33601/2001 (H3N2)
 Sw/Spain/54008/04 (H3N2)
 Sw/Spain/39139/02 (H3N2)
 Sw/Spain/42386/02 (H3N2)
 Sw/Spain/50047/03 (H1N1)
 Sw/Italy/1521/98 (H1N2)
 Sw/Cotes dArmor/790/97 (H1N2)
 Sw/Cotes dArmor/2433/98 (H1N2)
 Sw/Cotes dArmour/3633/84 (H3N2)
 Sw/Finistere/2899/82 (H1N1)
 Sw/Virginia/671/87 (H1N1)
 Sw/Virginia/670/87 (H1N1)
 Sw/Nakhon pathom/NIAH586-1/05 (H3N2)
 Sw/Chonburi/NIAH977/04 (H1N1)
 Sw/Chonburi/NIAH9469/04 (H1N1)
 Thailand/271/05 (H1N1)
 Sw/Chachoengsao/NIAH587/05 (H1N1)
 Sw/Chonburi/NIAH589/05 (H1N1)
 ● Sw/Ratchaburi/NIAH101942/08 (H1N1)
 ● Sw/Saraburi/NIAH116625-11/09 (H1N1)
 ● Sw/Saraburi/NIAH100761-22/09 (H1N1)
 goose/Leipzig/187-7/1979 (H7N7)
Cluster ALa
Human, avian, swine viruses
Cluster ALb
Supplementary figure 2. Phylogenetic tree of PA genes of Eurasian avian-like swine lineage. SIVs isolated in this study are indicated by ●.　ALa and ALb clusters in the Thai isolates are shown. Sw represents swine.
0.02

## Slide 3
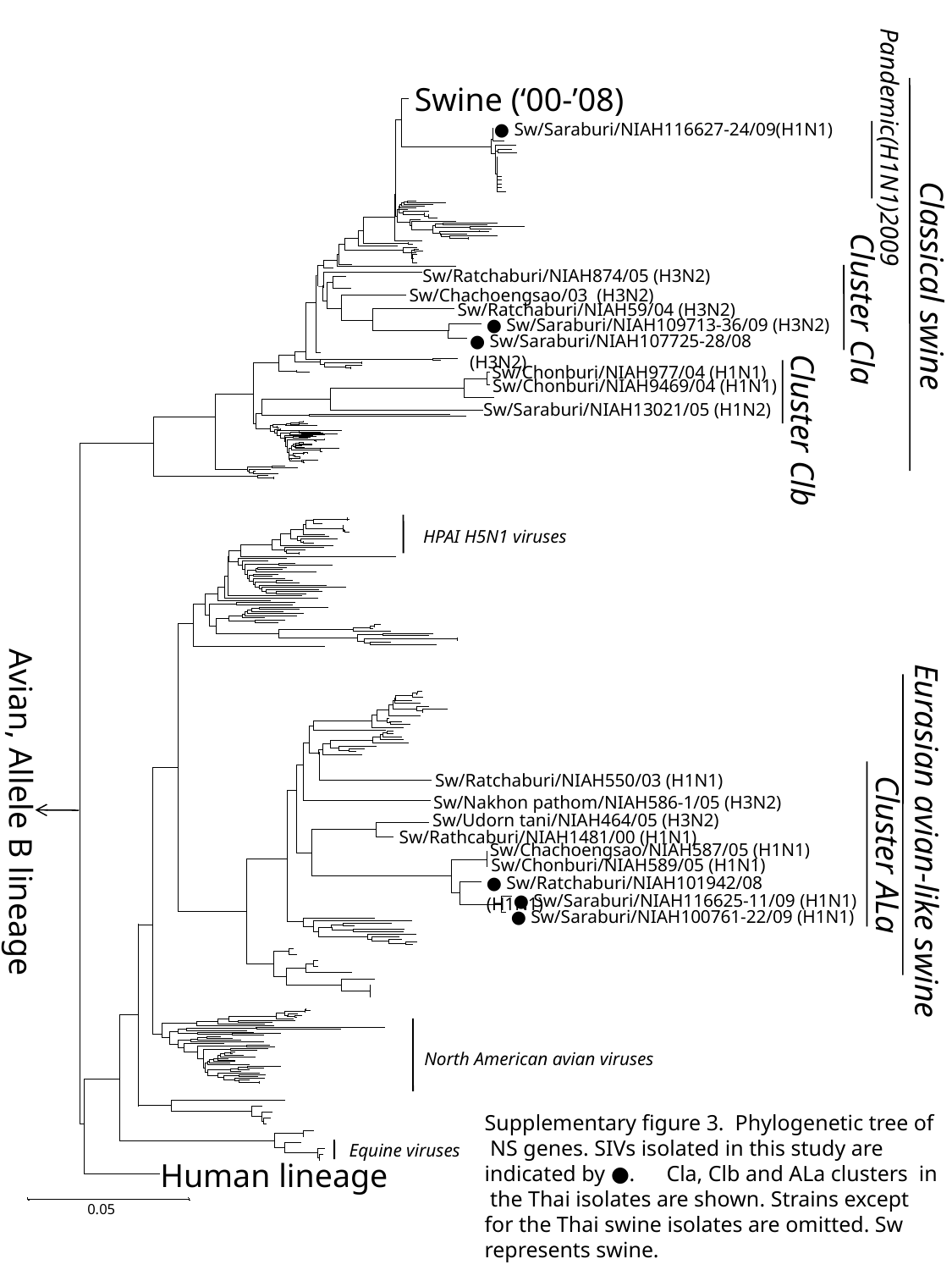

Swine (‘00-’08)
● Sw/Saraburi/NIAH116627-24/09(H1N1)
Pandemic(H1N1)2009
Classical swine
Cluster Cla
Sw/Ratchaburi/NIAH874/05 (H3N2)
Sw/Chachoengsao/03 (H3N2)
Sw/Ratchaburi/NIAH59/04 (H3N2)
● Sw/Saraburi/NIAH109713-36/09 (H3N2)
● Sw/Saraburi/NIAH107725-28/08 (H3N2)
Cluster Clb
Sw/Chonburi/NIAH977/04 (H1N1)
Sw/Chonburi/NIAH9469/04 (H1N1)
Sw/Saraburi/NIAH13021/05 (H1N2)
HPAI H5N1 viruses
Eurasian avian-like swine
Sw/Ratchaburi/NIAH550/03 (H1N1)
Cluster ALa
Sw/Nakhon pathom/NIAH586-1/05 (H3N2)
Avian, Allele B lineage
Sw/Udorn tani/NIAH464/05 (H3N2)
Sw/Rathcaburi/NIAH1481/00 (H1N1)
Sw/Chachoengsao/NIAH587/05 (H1N1)
Sw/Chonburi/NIAH589/05 (H1N1)
● Sw/Ratchaburi/NIAH101942/08 (H1N1)
● Sw/Saraburi/NIAH116625-11/09 (H1N1)
● Sw/Saraburi/NIAH100761-22/09 (H1N1)
North American avian viruses
Supplementary figure 3. Phylogenetic tree of NS genes. SIVs isolated in this study are indicated by ●.　Cla, Clb and ALa clusters in the Thai isolates are shown. Strains except for the Thai swine isolates are omitted. Sw represents swine.
Equine viruses
Human lineage
0.05

## Slide 4
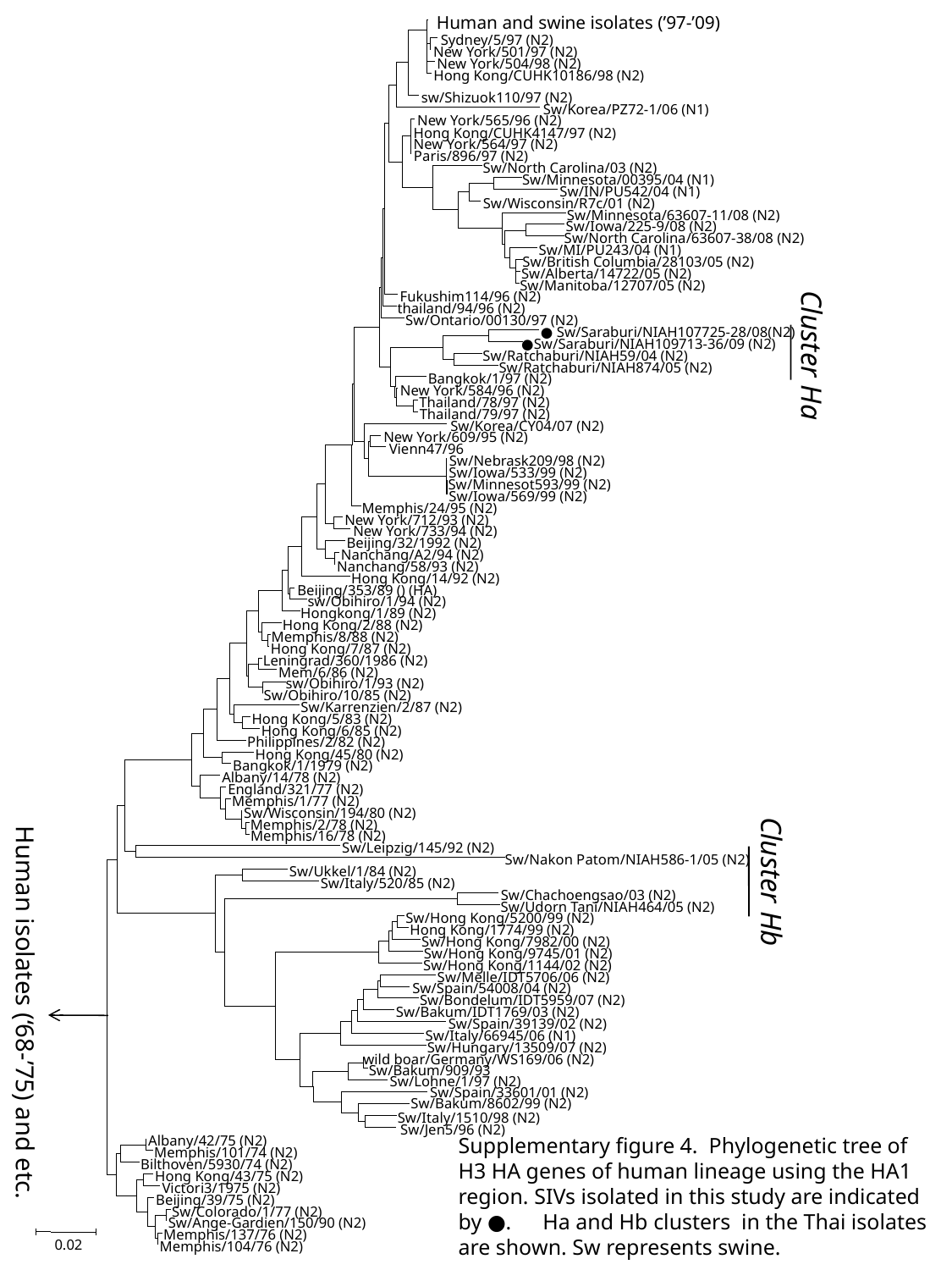

Human and swine isolates (’97-’09)
 Sydney/5/97 (N2)
 New York/501/97 (N2)
 New York/504/98 (N2)
 Hong Kong/CUHK10186/98 (N2)
 sw/Shizuok110/97 (N2)
 Sw/Korea/PZ72-1/06 (N1)
 New York/565/96 (N2)
 Hong Kong/CUHK4147/97 (N2)
 New York/564/97 (N2)
 Paris/896/97 (N2)
 Sw/North Carolina/03 (N2)
 Sw/Minnesota/00395/04 (N1)
 Sw/IN/PU542/04 (N1)
 Sw/Wisconsin/R7c/01 (N2)
 Sw/Minnesota/63607-11/08 (N2)
 Sw/Iowa/225-9/08 (N2)
 Sw/North Carolina/63607-38/08 (N2)
 Sw/MI/PU243/04 (N1)
 Sw/British Columbia/28103/05 (N2)
 Sw/Alberta/14722/05 (N2)
 Sw/Manitoba/12707/05 (N2)
● Sw/Saraburi/NIAH107725-28/08(N2)
 ●Sw/Saraburi/NIAH109713-36/09 (N2)
 Sw/Ratchaburi/NIAH59/04 (N2)
 Sw/Ratchaburi/NIAH874/05 (N2)
 Bangkok/1/97 (N2)
 New York/584/96 (N2)
 Thailand/78/97 (N2)
 Thailand/79/97 (N2)
 Sw/Korea/CY04/07 (N2)
 New York/609/95 (N2)
 Vienn47/96
 Sw/Nebrask209/98 (N2)
 Sw/Iowa/533/99 (N2)
 Sw/Minnesot593/99 (N2)
 Sw/Iowa/569/99 (N2)
 Memphis/24/95 (N2)
 New York/712/93 (N2)
 New York/733/94 (N2)
 Beijing/32/1992 (N2)
 Nanchang/A2/94 (N2)
 Nanchang/58/93 (N2)
 Hong Kong/14/92 (N2)
 Beijing/353/89 () (HA)
 sw/Obihiro/1/94 (N2)
 Hongkong/1/89 (N2)
 Hong Kong/2/88 (N2)
 Memphis/8/88 (N2)
 Hong Kong/7/87 (N2)
 Leningrad/360/1986 (N2)
 Mem/6/86 (N2)
 sw/Obihiro/1/93 (N2)
 Sw/Obihiro/10/85 (N2)
 Sw/Karrenzien/2/87 (N2)
 Hong Kong/5/83 (N2)
 Hong Kong/6/85 (N2)
 Philippines/2/82 (N2)
 Hong Kong/45/80 (N2)
 Bangkok/1/1979 (N2)
 Albany/14/78 (N2)
 England/321/77 (N2)
 Memphis/1/77 (N2)
 Sw/Wisconsin/194/80 (N2)
 Memphis/2/78 (N2)
 Memphis/16/78 (N2)
 Sw/Leipzig/145/92 (N2)
 Sw/Nakon Patom/NIAH586-1/05 (N2)
 Sw/Ukkel/1/84 (N2)
 Sw/Italy/520/85 (N2)
 Sw/Chachoengsao/03 (N2)
 Sw/Udorn Tani/NIAH464/05 (N2)
 Sw/Hong Kong/5200/99 (N2)
 Hong Kong/1774/99 (N2)
 Sw/Hong Kong/7982/00 (N2)
 Sw/Hong Kong/9745/01 (N2)
 Sw/Hong Kong/1144/02 (N2)
 Sw/Melle/IDT5706/06 (N2)
 Sw/Spain/54008/04 (N2)
 Sw/Bondelum/IDT5959/07 (N2)
 Sw/Bakum/IDT1769/03 (N2)
 Sw/Spain/39139/02 (N2)
 Sw/Italy/66945/06 (N1)
 Sw/Hungary/13509/07 (N2)
 wild boar/Germany/WS169/06 (N2)
 Sw/Bakum/909/93
 Sw/Lohne/1/97 (N2)
 Sw/Spain/33601/01 (N2)
 Sw/Bakum/8602/99 (N2)
 Sw/Italy/1510/98 (N2)
 Sw/Jen5/96 (N2)
 Fukushim114/96 (N2)
 thailand/94/96 (N2)
 Sw/Ontario/00130/97 (N2)
Cluster Ha
Cluster Hb
Human isolates (‘68-’75) and etc.
Supplementary figure 4. Phylogenetic tree of H3 HA genes of human lineage using the HA1 region. SIVs isolated in this study are indicated by ●.　Ha and Hb clusters in the Thai isolates are shown. Sw represents swine.
 Albany/42/75 (N2)
 Memphis/101/74 (N2)
 Bilthoven/5930/74 (N2)
 Hong Kong/43/75 (N2)
 Victori3/1975 (N2)
 Beijing/39/75 (N2)
 Sw/Colorado/1/77 (N2)
 Sw/Ange-Gardien/150/90 (N2)
 Memphis/137/76 (N2)
0.02
 Memphis/104/76 (N2)
